# Supplementary material for: Development of a computer-aided design software for the quantitative evaluation of aesthetic damage
Source: PLoS One. 2019 Dec 18;14(12):e0226322. doi: 10.1371/journal.pone.0226322 (PMC6919621; doi:10.1371/journal.pone.0226322)
Supplement: S1 Code site — 1. (ZIP) [file pone.0226322.s001.zip › [EESC_jr][Projeto]Relatorio_07_21/reports/templates_zip/backyard-high-converting-landing-page-template.zip!/backyard/backyard/shortcodes.html]

Backyard Landing Page


## Shortcodes / Typography

Toggle navigation

- Basic
- Button
- Icon
- Alert
- Table
- Column

Lorem ipsum dolor sit amet, consectetur text link elit, sed do eiusmod tempor incididunt ut labore et dolore magna aliqua. Ut enim *ad minim veniam, quis nostrud exercitatio ullamcon* laboris nisi ut aliquip ex ea commodo consequat.

# H1 Heading

Lorem ipsum dolor sit amet, consectetur elit, sed do eiusmod tempor incididunt sed do eiusmod tempor incididunt ut labore et dolore magna aliqua.

## H2 Heading

Lorem ipsum dolor sit amet, consectetur elit, sed do eiusmod tempor incididunt sed do eiusmod tempor incididunt ut labore et dolore magna aliqua.

### H3 Heading

Lorem ipsum dolor sit amet, consectetur elit, sed do eiusmod tempor incididunt sed do eiusmod tempor incididunt ut labore et dolore magna aliqua.

Lorem ipsum dolor sit amet, consectetur highlighted elit, sed do eiusmod tempor incididunt ut labore et dolore magna aliqua. Ut enim ad minim veniam, **bold text** laboris nisi ut aliquip ex ea commodo consequat.

#### H4 Heading

Lorem ipsum dolor sit amet, consectetur elit, sed do eiusmod tempor incididunt sed do eiusmod tempor incididunt ut labore et dolore magna aliqua.

##### H5 Heading

Lorem ipsum dolor sit amet, consectetur elit, sed do eiusmod tempor incididunt sed do eiusmod tempor incididunt ut labore et dolore magna aliqua.

###### H6 Small Paragraph

Lorem ipsum dolor sit amet, consectetur elit, sed do eiusmod tempor incididunt sed do eiusmod tempor incididunt ut labore et dolore magna aliqua.

### BUTTON

- Default
- Primary
- Secondary

### SOCIAL ICONS

### CONTACT FORM

×
**Success Alert.** You successfully read this important alert message.

×
**Info Alert.** This alert needs your attention, but it's not super important.

×
**Warning Alert.** Better check yourself, you're not looking too good.

×
**Danger Alert.** Change a few things up and try submitting again.

| Package | Details | Price |
| --- | --- | --- |
| Basic | 100MB Disk Space | **$9** |
| Standard | 400MB Bandwidth | **$19** |
| Advance | 10 Subdomains | **$29** |
| Ultimate | 50 Email Accounts | **$49** |
| Professional | 24/7 Support | **$99** |

##### 1/1

Lorem ipsum dolor sit amet, consectetur elit, sed do eiusmod tempor incididunt sed do eiusmod tempor incididunt ut labore et dolore magna aliqua eiusmod tempor incididunt sed do eiusmod tempor incididunt ut labore et dolore magna aliqua.

##### 1/2

Lorem ipsum dolor sit amet, consectetur elit, sed do eiusmod tempor incididunt sed do eiusmod tempor incididunt ut labore et dolore magna aliqua eiusmod tempor.

##### 1/2

Lorem ipsum dolor sit amet, consectetur elit, sed do eiusmod tempor incididunt sed do eiusmod tempor incididunt ut labore et dolore magna aliqua eiusmod tempor.

##### 2/3

Lorem ipsum dolor sit amet, consectetur elit, sed do eiusmod tempor incididunt sed do eiusmod tempor incididunt ut labore et dolore magna aliqua eiusmod tempor.

##### 1/3

Lorem ipsum dolor sit amet, consectetur elit, sed do eiusmod tempor incididunt sed do eiusmod tempor incididunt ut labore.

##### 1/3

Lorem ipsum dolor sit amet, consectetur elit, sed do eiusmod tempor incididunt sed do eiusmod tempor incididunt ut labore.

##### 1/3

Lorem ipsum dolor sit amet, consectetur elit, sed do eiusmod tempor incididunt sed do eiusmod tempor incididunt ut labore.

##### 1/3

Lorem ipsum dolor sit amet, consectetur elit, sed do eiusmod tempor incididunt sed do eiusmod tempor incididunt ut labore.

##### 3/4

Lorem ipsum dolor sit amet, consectetur elit, sed do eiusmod tempor incididunt sed do eiusmod tempor incididunt ut labore et dolore magna aliqua eiusmod tempor. Lorem ipsum dolor sit amet, consectetur elit, sed do eiusmod tempor incididunt sed do eiusmod tempor incididunt ut labore et dolore magna aliqua eiusmod tempor.

##### 1/4

Lorem ipsum dolor sit amet, consectetur elit, sed do eiusmod tempor incididunt.

##### 1/4

Lorem ipsum dolor sit amet, consectetur elit, sed do eiusmod tempor incididunt.

##### 1/4

Lorem ipsum dolor sit amet, consectetur elit, sed do eiusmod tempor incididunt.

##### 1/4

Lorem ipsum dolor sit amet, consectetur elit, sed do eiusmod tempor incididunt.

##### 1/4

Lorem ipsum dolor sit amet, consectetur elit, sed do eiusmod tempor incididunt.


---

Copyright Backyard 2015
